# Patch Dehiscence After Mitral Valve Repair by Posterior Mitral Leaflet Augmentation at Weaning From Cardiopulmonary Bypass

**DOI:** 10.7759/cureus.62872

**Published:** 2024-06-21

**Authors:** Satoshi Aihara, Yoshinobu Nakayama, Yasufumi Nakajima, Takatoshi Tsujimoto, Koichi Akiyama

**Affiliations:** 1 Anesthesiology and Critical Care, Kansai Medical University, Osaka, JPN; 2 Anesthesiology and Critical Care, Kindai University, Osaka, JPN

**Keywords:** three-dimensional echocardiography, transesophageal echocardiography, cardiac surgical procedures, myocardial ischemia, mitral valve insufficiency

## Abstract

The treatment for ischemic mitral regurgitation (MR) involves a combination of medical management and, in certain cases, surgical intervention. The approach depends on the severity of the condition, underlying causes, and the patient's overall health. A 76-year-old male with heart failure refractory to medical management because of ischemic MR was considered for a mitral valve repair surgery with a posterior mitral leaflet augmentation technique. Following the repair of the mitral valve and the cessation of the initial cardiopulmonary bypass (CPB), a prolapse of the posterior mitral leaflet, which had not been detected before the surgery, was revealed by intraoperative transesophageal echocardiography (TEE). A thorough inspection of the repaired mitral valve after cardiac arrest during the second CPB unveiled a loose suture at the edge of the valve and an inverted pericardium, indicating that the patch had flipped upward. Although complications of this type following the augmentation of the mitral posterior leaflet are rare, we promptly detected it using TEE, which incorporates three-dimensional imaging.

## Introduction

The treatment of ischemic mitral regurgitation (MR) primarily involves medication and lifestyle changes. In the case of valvular procedures, the decision between mitral valve plasty (MVP) and mitral valve replacement (MVR) in ischemic MR is tailored to the individual patient's condition [1]. Although the exact surgical technique of MVP aimed at improving overall outcomes for patients with ischemic MR has not been clearly outlined, recent reports have highlighted excellent early patient results with the extended posterior leaflet augmentation technique, which facilitates deep leaflet coaptation [2].

## Case presentation

The patient’s written consent was obtained for data collection and anonymous publication. A 76-year-old man with medically refractory heart failure due to ischemic MR was scheduled to undergo MVP. Four years prior, percutaneous coronary intervention was performed on the anterior descending artery for unstable angina. Preoperative transthoracic echocardiography revealed a dilated left atrium (47 mm), left ventricular diastolic/systolic dimensions (55/46 mm), and impaired contraction with an ejection fraction of 31% (modified Simpson’s method), including diffuse wall hypokinesis. The MR was severe, with an effective regurgitant orifice of 0.39 cm², regurgitant volume of 69 mL, and posterior leaflet tethering.

**Figure 1:**
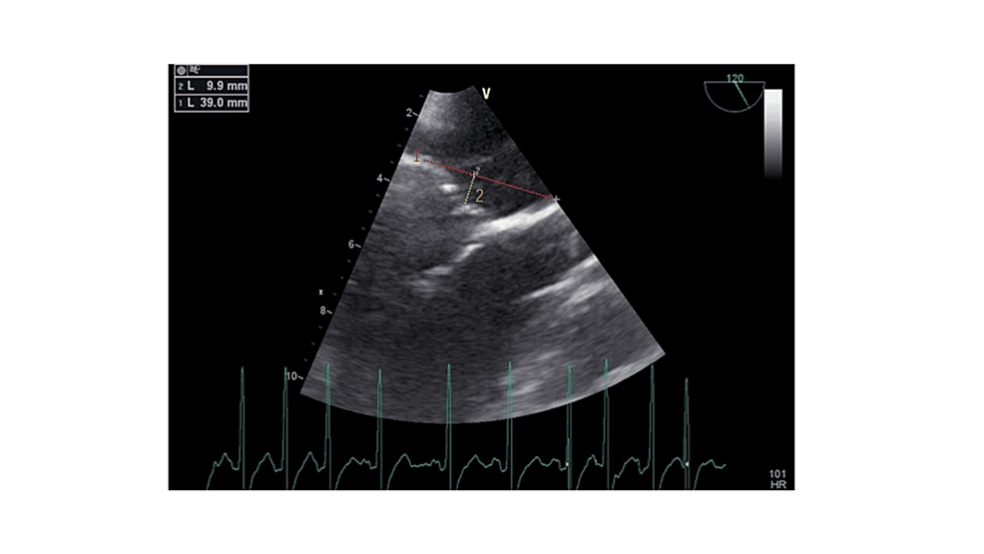
The patient’s TEE image before CPB The image reveals a dilated mitral annulus with a diameter of 39 mm (red line), loss of mitral valve coaptation between A2 and P2, and tethering in the posterior leaflet with a height of 9.9 mm (yellow line). TEE, transesophageal echocardiography; A2, middle segment of the anterior mitral leaflet; P2, middle scallop of the posterior mitral leaflet; CPB, cardiopulmonary bypass

Color-flow Doppler imaging in the pre-cardiopulmonary bypass (CPB) transesophageal electrocardiography (TEE) showed a severe central MR jet due to malcoaptation of the leaflets. The mid-esophageal long-axis, mitral commissural, and three-dimensional TEE views also showed a severe MR jet. The transgastric mid-short-axis view showed dilatation of the left ventricle with global hypokinesis (Figure 1; Video 1).

**Video 1:** The patient’s TEE movie before CPB TEE, transesophageal echocardiography; CPB, cardiopulmonary bypass

CPB was initiated by cannulation of the ascending aorta and superior and inferior vena cavae. Access to the left atrium was achieved via the superior transeptal approach, revealing thickening of both MV leaflets and calcification in the P2/P3 valve area. Rupture of the chordae tendineae was not observed. Owing to calcification between P2 and P3, the affected portion of the posterior leaflet was excised. After trimming, the autologous pericardium was sutured to the PML defect for augmentation.

**Figure 2:**
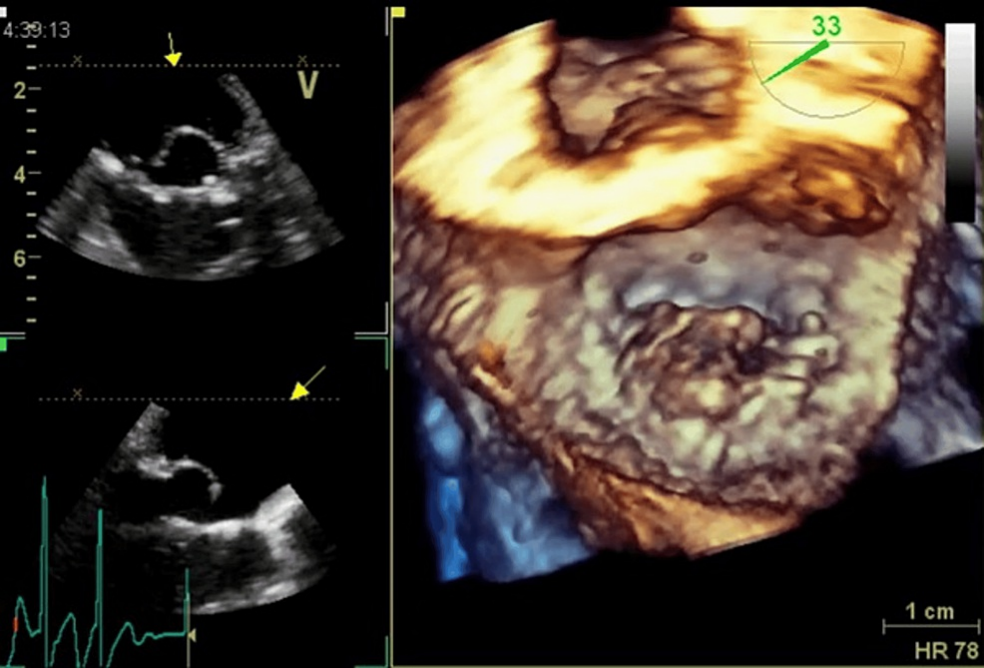
The patient’s TEE image after the termination of the first CPB Three-dimensional TEE image displays a prolapse complication in the P1 to P2 segments of the mitral valve, areas that have been reinforced with pericardial tissue. TEE, transesophageal echocardiography; P1, anterior scallop of the posterior mitral leaflet; P2, middle scallop of the posterior mitral leaflet; CPB, cardiopulmonary bypass

Upon discontinuation of CPB, TEE showed no evidence of MR; however, follow-up TEE at 5 min after CPB discontinuation revealed an anteriorly directed MR jet in the mid-esophageal long-axis view. Prolapse of the posterior mitral leaflet, which was not present immediately after CPB, was evident in both the mid-esophageal mitral commissural and long-axis views in the follow-up TEE, as seen in Video 2. Furthermore, three-dimensional TEE revealed a flaw in the P1 and P2 segments of the MV, which was augmented with pericardial material (Figure 2, Video 2).

**Video 2:** The patient’s TEE movie after the termination of the first CPB TEE, transesophageal echocardiography; CPB, cardiopulmonary bypass

**Figure 3:**
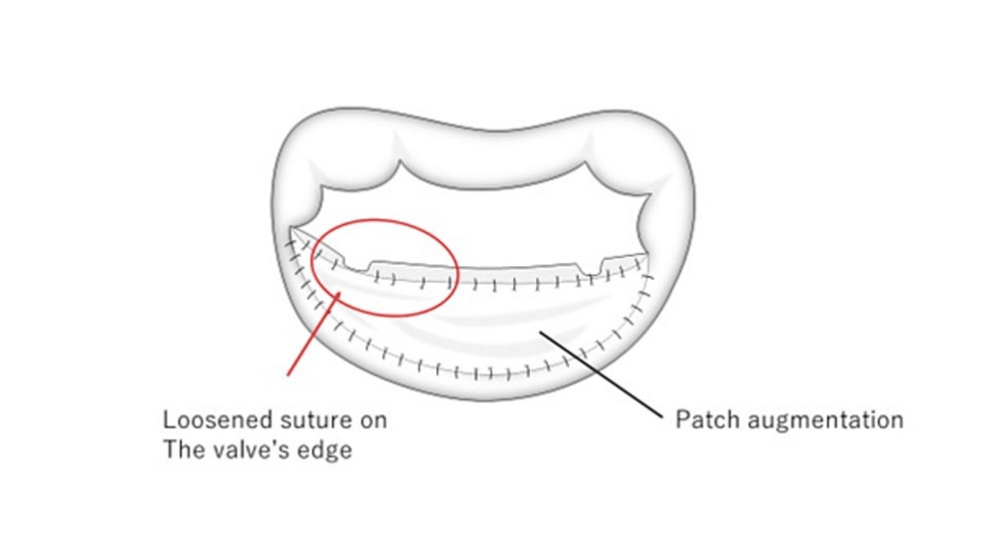
Illustrations of a surgical procedure Illustrations of a surgical procedure show the augmentation of the mitral posterior leaflet and the dehiscent area in this specific case. This illustration is created by the authors themselves.

CPB was subsequently reinitiated when a direct examination of the repaired MV revealed a loosened suture on the valve edge and an upturned pericardium, signifying detachment of the patch. The autologous pericardium was reattached at the loosened site of the posterior mitral leaflet (Figure 3). The adequacy of leaflet coaptation was verified, showing no residual regurgitation after termination of the second CPB, as documented in Video 3.

**Video 3:** The patient’s TEE movie after the termination of the second CPB TEE, transesophageal echocardiography; CPB, cardiopulmonary bypass

## Discussion

Ischemic MR, a serious complication that can occur after myocardial infarction, is classified as a Carpentier type IIIb valvular dysfunction. This condition primarily occurs due to alterations in the MV leaflets caused by enlargement and reduced contractility of the left ventricle. These changes have a significant negative effect on patient prognosis. Approximately 20-30% of patients with ischemic heart conditions develop functional MR. Surgical intervention is considered for these patients if they meet specific hemodynamic severity criteria and continue to show symptoms of heart failure despite receiving optimal medical treatment. Surgical approaches for addressing ischemic MR include MVP and MVR [3,4]. Various techniques, such as ring annuloplasty, edge-to-edge method, and mitral leaflet augmentation, are employed, sometimes in conjunction with repairs to the subvalvular apparatus. Despite these options, the most appropriate surgical technique for the treatment of ischemic MR remains a topic of ongoing debate [5].

Mitral leaflet augmentation, used to treat ischemic MR, involves using extra materials, such as autologous pericardial patches. This method is particularly necessary in severe cases of MR caused by conditions such as infective endocarditis, rheumatic or congenital diseases, or in instances of recurrent MR following repair procedures for degenerative diseases. The purpose of this technique is to create an adequate surface for coaptation. Numerous studies have documented the rate of avoiding reoperation under particular circumstances. One study reported five-year and 10-year rates of 94±3% and 82±7%, respectively [6,7]. Another study reported a five-year rate of 95.1%. Furthermore, a notable disparity was observed in the rates of freedom from reoperation between cases with and without leaflet augmentation. Specifically, the rates at five years were 96.2±2.2% versus 93.4±3.2%, and at 10 years, the rates were 89.7±4.5% versus 68.8±13.7% in cases with and without augmentation, respectively (log-rank, P=0.008) [8].

Other authors have reported similar accidents. Early technical failures have been noted, such as augmentation of the anterior mitral leaflet (AML), which detaches the pericardial patch at the lower anterolateral section of the AML [9]. Additionally, a separate group of authors has described a case of acute MR due to patch detachment from the anastomosis after PML augmentation, which led to severe complications one week after discharge. In this case, necrosis of the remaining PML rendered further repair unfeasible. Using autologous pericardial patches for MV repair can cause long-term complications, such as mitral stenosis, recurrent MR, and detachment or tearing of the pericardial patch. Intraoperative TEE, including three-dimensional TEE, is invaluable for assessing the causes of MR and the functionality of the MV after repair [10].

In the present case, the patient was resistant to medical treatment. Severe MR results from ischemic heart disease causes left ventricular dilation and leaflet tethering. Owing to heavy calcification in the body of the PML, PML augmentation was performed. The majority of the calcified section of the PML was removed, and the defect was repaired using an autologous pericardium. Intraoperative patch detachment was observed. Continuous TEE monitoring can detect early abnormal valve motion and prevent various MV dysfunctions during and after surgery.

## Conclusions

Patch detachment after MV repair with PML augmentation is rare but can occur. This case highlights the importance of intraoperative TEE, particularly during CPB weaning, in patients undergoing MV repair. TEE imaging facilitates monitoring the repair process and repaired area, preventing serious complications. Additionally, three-dimensional TEE imaging revealed the motion of the repaired leaflet and visualized abnormal patch motion.

## References

[1] Mohananey D, Aljadah M, Smith AA, Haines JF, Patel S, Villablanca P, Ramakrishna H (2022). The 2020 ACC/AHA Guidelines for Management of Patients With Valvular Heart Disease: Highlights and Perioperative Implications. J Cardiothorac Vasc Anesth.

[2] Ikeda N, Yamaguchi H, Takagaki M (2019). Extended Posterior Leaflet Augmentation for Ischemic Mitral Regurgitation　- Augmented Posterior Leaflet Snuggling up to Anterior Leaflet. Circ J.

[3] Salmasi MY, Acharya M, Humayun N, Baskaran D, Hubbard S, Vohra H (2016). Is valve repair preferable to valve replacement in ischaemic mitral regurgitation? A systematic review and meta-analysis. Eur J Cardiothorac Surg.

[4] Sharma A, Agrawal S, Goel S, Borer JS (2017). Surgical Treatment of Ischemic Mitral Regurgitation: Valve Repair Versus Replacement. Curr Cardiol Rep.

[5] Nappi F, Antoniou GA, Nenna A (2022). Treatment options for ischemic mitral regurgitation: A meta-analysis. J Thorac Cardiovasc Surg.

[6] Shomura Y, Okada Y, Nasu M (2013). Late results of mitral valve repair with glutaraldehyde-treated autologous pericardium. Ann Thorac Surg.

[7] Ng CK, Nesser J, Punzengruber C, Pachinger O, Auer J, Franke H, Hartl P (2001). Valvuloplasty with glutaraldehyde-treated autologous pericardium in patients with complex mitral valve pathology. Ann Thorac Surg.

[8] Fukunaga N, Sakata R, Koyama T (2017). Reoperative analysis after mitral valve repair with glutaraldehyde-treated autologous pericardium. Interact Cardiovasc Thorac Surg.

[9] Mihos CG, Horvath SA, Fernandez R, Nappi F, Xydas S (2020). Early failure of mitral valve repair with anterior leaflet pericardial patch augmentation in rheumatic and radiation-induced valvulitis. J Thorac Dis.

[10] Gomibuchi T, Takano T, Wada Y, Terasaki T, Seto T, Fukui D (2015). Patch detachment after mitral valve repair with posterior leaflet augmentation: a case report. J Cardiothorac Surg.

